# High-resolution spectral information enables phenotyping of leaf epicuticular wax in wheat

**DOI:** 10.1186/s13007-021-00759-w

**Published:** 2021-06-07

**Authors:** Fátima Camarillo-Castillo, Trevis D. Huggins, Suchismita Mondal, Matthew P. Reynolds, Michael Tilley, Dirk B. Hays

**Affiliations:** 1 Global Wheat Program, International Maize and Wheat Improvement Center (CIMMYT), Apdo. Postal 6-641, Mexico, D.F 06600 Mexico; 2 USDA ARS, Dale Bumper National Rice Research Center, Stuttgart, AR 72160 USA; 3 Agricultural Research Service, Center for Grain and Animal Health Research, USDA, 1515 College Ave., Manhattan, KS 66502 USA; 4 Department of Soil and Crop Sciences, Texas A&M University, College Station, Texas, 77840 USA

**Keywords:** Plant cuticle, Vegetation indices, High-throughput phenotyping, Wheat breeding

## Abstract

**Background:**

Epicuticular wax (EW) is the first line of defense in plants for protection against biotic and abiotic factors in the environment. In wheat, EW is associated with resilience to heat and drought stress, however, the current limitations on phenotyping EW restrict the integration of this secondary trait into wheat breeding pipelines. In this study we evaluated the use of light reflectance as a proxy for EW load and developed an efficient indirect method for the selection of genotypes with high EW density.

**Results:**

Cuticular waxes affect the light that is reflected, absorbed and transmitted by plants. The narrow spectral regions statistically associated with EW overlap with bands linked to photosynthetic radiation (500 nm), carotenoid absorbance (400 nm) and water content (~ 900 nm) in plants. The narrow spectral indices developed predicted 65% (EWI-13) and 44% (EWI-1) of the variation in this trait utilizing single-leaf reflectance. However, the normalized difference indices EWI-4 and EWI-9 improved the phenotyping efficiency with canopy reflectance across all field experimental trials. Indirect selection for EW with EWI-4 and EWI-9 led to a selection efficiency of 70% compared to phenotyping with the chemical method. The regression model EWM-7 integrated eight narrow wavelengths and accurately predicted 71% of the variation in the EW load (mg·dm^−2^) with leaf reflectance, but under field conditions, a single-wavelength model consistently estimated EW with an average RMSE of 1.24 mg·dm^−2^ utilizing ground and aerial canopy reflectance.

**Conclusions:**

Overall, the indices EWI-1, EWI-13 and the model EWM-7 are reliable tools for indirect selection for EW based on leaf reflectance, and the indices EWI-4, EWI-9 and the model EWM-1 are reliable for selection based on canopy reflectance. However, further research is needed to define how the background effects and geometry of the canopy impact the accuracy of these phenotyping methods.

## Background

Wheat is a major staple food and an important source of calories in developing countries [1]. More than 220 million ha of wheat is cultivated worldwide [2], and 600 million tons of wheat grain is produced each year [3]. The expected global population of 9 billion by 2050 will require an increase in wheat production of 60% to 100% [2, 4], but the current genetic gains of < 1% per year [5] will be insufficient to fulfill this expected demand. Annually, more than 600 million tons of wheat are harvested [6], but maintaining this production is already a challenge in the face of climate change. It is estimated that climate change can reduce global wheat production by 6% for every degree centigrade increase in the temperature [7]. Therefore, the development of wheat cultivars that are adapted to high temperatures and limited irrigation is crucial for responding to a changing climate and ensuring wheat production.

Developing wheat cultivars that are adapted to a wide range of environments, have resilience to abiotic stresses and high yield potential are priorities of the main public breeding programs [2]. Physiological trait-based improvements for tolerance to heat and drought stress rely on the favorable expression of morphological and physiological plant traits (PT) [8–10]. Independent conceptual models for grain yield (GY) under heat and drought have been proposed based on the following main drivers: light interception (LI), radiation use efficiency (RUE), partitioning of total assimilates [8], water use efficiency (WUE) and harvest index [11]. Each of these main drivers contains genetically determined PTs that can potentially lead to an additive genetic effect for resilience to heat and drought when combined through strategic crossing [12, 13]. Physiological traits such as canopy temperature (CT) are already utilized as selection criteria in breeding pipelines [5, 10], but key PTs such as epicuticular wax (EW) remain unexplored because of the expensive, subjective and laborious method for phenotyping [14].

EW is the outermost layer of leaves that is located on the top of the cutin matrix and intracuticular wax [15] and consist of hydrocarbon compounds [16, 17] derived from long chains of C_20_ and C_30_ fatty acids [18, 19]. The hydrophobic layer that creates the EW covers the aerial epidermis of plants maintaining the integrity of the plant against high UV radiation [20] and external environmental stresses such as insect infestation [21, 22] and pathogen infection [23]. This cuticle also minimizes the water loss via transpiration in wheat [18, 24] and reduces leaf temperature [25, 26]. Early studies estimated a decrease in the internal temperature of the plant by 0.7° C under simulated drought conditions in a glasshouse, saving 30 g of water per plant during the growth season and extending grain filling by 3 days [27].

Waxes and trichomes affect the interaction of the plant with the environment, particularly the reflection and absorbance of light. Surface waxes are very effective in reflecting excessive radiation in specific ranges of the spectrum, namely at 330 and 680 nm [28], but the main increases in radiation reflection occur at the photosynthetic active radiation (PAR) range to dissipate excess energy and avoid damage to the PSII reaction center [29, 30]. In wheat, increases in light reflectance of 12% to 35% were detected at the PAR (400 to 700 nm) range in wax covered genotypes [31]. Several studies have reported that EW and its constituents are an important protective barrier against harmful UV-B radiation [20, 28, 32–35], but these fluctuations in reflectance are species-specific and can range from < 10% in most species to 70% in others [21, 36].

Limitations on field phenotyping restrict our capacity to unravel complex morphological and physiological traits. Spectral technologies have the potential to increase the efficiency, precision and accuracy of phenotyping platforms. In breeding programs, high-precision phenotyping can enable the screening of segregating material, advanced lines and germplasm [5, 37]. Increasing the accuracy of phenotyping can provide more reliable estimates of heritability and variance components [38], facilitate gene discovery and enable prediction of complex traits with approaches such as genomic selection [39, 40]. The strong association of spectral secondary traits with GY [41, 42] highlights the potential of canopy reflectance to increase productivity in wheat. A detailed list of sensors and their applications for plant phenotyping is provided by [43].

Recent advances in technology have maximized the throughput of phenotyping measurements under field and controlled conditions [44–46]. RGB and hyperspectral sensors have enabled the rapid and noninvasive acquisition of spectral information. Spectral vegetation indices (SVI) are a quick, easy and inexpensive method of transforming light reflectance into simple indicators of photosynthetic and canopy variations. The simple ratio index (SR) [47] and the normalized difference vegetation index (NDVI) [48] are two of the first SVIs developed for detecting green vegetation. Both indices combine the percentage of reflectance at the wavelengths where plants absorb (~ 750 to 800 nm) and reflect (800 to 2500 nm) light. Several other SVIs have been built for sensing the water content of plants [49], photosynthetic radiation [50], carotenoid pigments [51], plant height [52], leaf area [53], and diseases [54].

In this study, the aim was to develop spectral methods to indirectly phenotype EW accumulated on the surface of leaves. This wax index will serve as a proxy to detect genotypes with a thick wax cover, in order to integrate the trait into breeding pipelines to enhance resilience to heat and drought stress. The goal is to facilitate frequent screening for EW at multiple field trial locations by replacing conventional sample-based methods. Additionally, these methods will support ongoing research on the underlaying physiological and genetic mechanisms of cuticular waxes. We conducted a set of theoretical studies with the following specific objectives: i) detect the wavelengths at which reflectance is affected by cuticular waxes, ii) develop spectral indices and models to detect wheat cultivars with high and low EW content, and iii) validate the spectral methods for phenotyping under field conditions.

## Results

### Light interactions associated with leaf EW

**Fig. 1:**
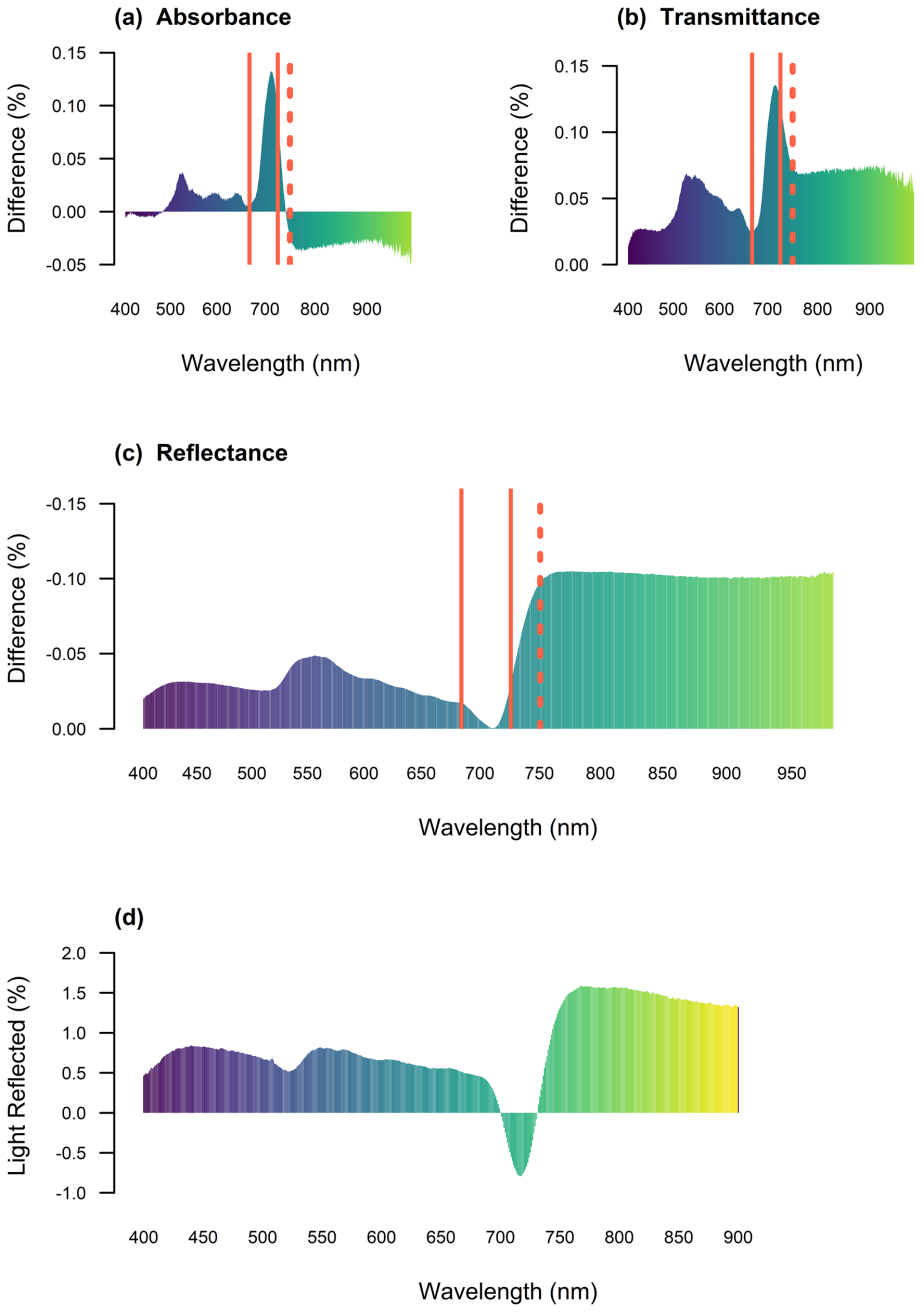
Variation in **a** absorbance, **b** transmittance and **c** reflectance derived by the removal of the EW coat with HPLC chloroform (CHCl3). These variations are presented as the difference of the spectral signature of the leaf after the wax coat was removed minus the the spectral response of the leaf with the wax coat in place. The red solid lines define the red edge and the dash line mark the end of the visible and start of the NIR region. The slope of the linear regression models (**d**) were fitted as *Y* = *a* + *bX*, where *Y* corresponds to the independent variable EW (mg·dm^−2^), *X* is the percentage of light reflectance at one nanometer resolution, *a* and *b* are the intercept and the slope of the fitted model, respectively. The statistical significance of the models was $P\le 0.05$ or less

The differences in the light interactions detected after the removal of EW confirmed the role of the cuticle in avoiding and dissipating excess radiation (Fig. 1). Variations in the percentage of light absorbed (Fig. 1a), transmitted (Fig. 1b) and reflected (Fig. 1c) by leaves were detected when EW was partially eliminated. The removal of the cuticle increased the light absorbance in the visible range from 0.02 to 0.04%, with a subsequent decrease to 0% reflectance at 710 nm and 0.03% in the NIR. An increase in light transmission through the leaf from 0.01 to 0.06% in the visible region was also observed, with a significant increment of 0.13% in the red-edge (680 to 740 nm). In the NIR (740 to 980 nm), the transmittance also increased by approximately 0.06%. Light reflectance was most affected when the wax cuticle was removed. Its removal revealed that EW contributed from 0.05% to 0.15% of the increase in reflectance at various wavelengths. Further analysis enabled the estimation of both positive and negative variations in the percentage of light reflected by the unit (mg·dm^2^) of wax accumulated on top of the leaf surface. Figure 1d presents the slopes of the linear regression models individually fitted with the EW content as the independent variable and the percentage of light reflectance detected with the spectroradiometer as the dependent variable. From 424 to 450 nm, there was an increase of ~ 0.82% in reflectance, and from 544 to 575 nm the increase was 0.79%. The light reflectance in the 700 and 730 nm was not affected by the cuticular wax; however, there was a reduction of 0.77% from 713 to 720 nm, with the highest peak in 717 nm (− 0.8%), and a subsequent increase of ~ 1.5% from 756 to 825 nm.

**Fig. 2:**
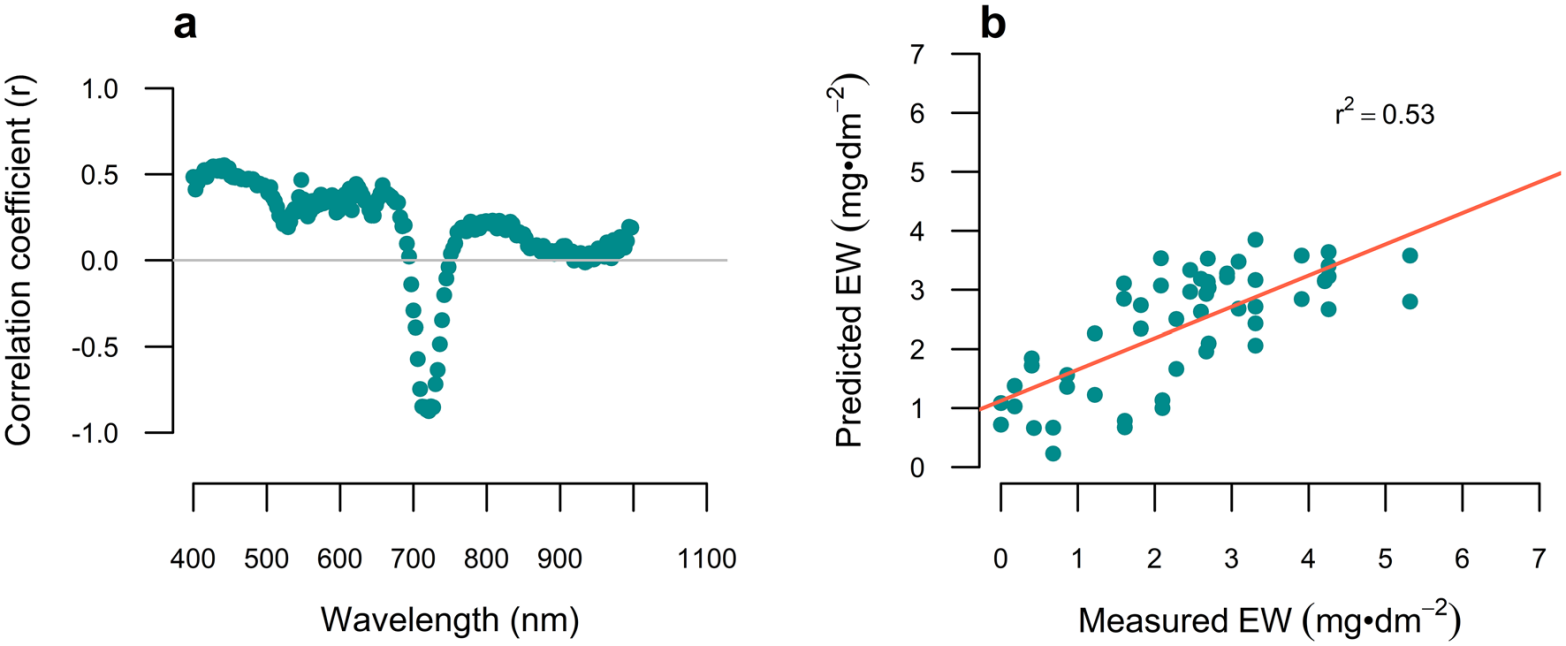
Correlation coefficients **a** of the three main partial least square components with the EW content (mg·dm^2^) and **b** association of the EW load predicted with the PLS’s model vs EW measured by the chemical method

The partial least square regression (PLSR) analysis reduced the total number of spectral bands and integrated only uncorrelated bands in the predictive model (Fig. 2). The correlation coefficients of the regression between the wavelengths and EW content are presented in Fig. 2a. Three main components enabled the maximum correlation between the wavelengths and the EW content and explained 97.34% of the variability of the trait. These three components combine the follow spectral regions: 424 to 448, 625, 660, 712 to 727, 775 to 835, 994 and 997 nm. Most of these wavelengths coincide with the regions detected in Fig. 1d. The most influential variables were 712 to 727 nm, where the transition from low reflectance in the visible region to high reflectance in the NIR wavelengths occurs. Overall, the selected latent variables or wavelengths enabled the prediction of EW content in the data subset for validation and lead to a positive linear association between the predicted and the actual values of EW (Fig. 2b).

### Spectral indices for indirect phenotyping of EW

Spectral indices developed in previous studies for indirect phenotyping of morphological and physiological characteristics of the plant (Tables 1 and 2) were calculated and statistically associated with EW. The spectral resolution of the light reflectance extracted from the spectroradiometer was adjusted from 1 to 3 nm by averaging the percentage of light reflectance of every 3 bands. The broad spectral bands were calculated by estimating the average of reflectance w ithin the range (nm) corresponding to the blue, green, yellow, orange, red, red-edge and near infrared regions. The ranges in nm of every spectral region are included in Table 2. The narrow spectral indices PRI-1 (r = −0.57), CARI-2 (r = − 0.67), PSSR-b (r = − 0.5 7), PSSR-a (r = − 0.55 ), ARI-1 (r = − 0.52) , ARI-2 (r = − 0.58) and SIPI-2 (− 0.61) were significantly correlated (*p* < *0.001*) with EW. These indices are effective to detect variations in carotenoids, chlorophyll and anthocyanins in plants [51, 55–58]. However, these indices were not able to predict more than 38% of the total variability of EW. Among the broad vegetation indices calculated, only RGRI (− 0.57) and ARI-1 (− 0.67) were strongly associated with EW.

**Table 1:** Narrow vegetation indices for phenotyping specific traits in plants

Narrow vegetation indices	Abbreviation	Formula	Reference
Water index	WI	${\rho }_{900} / {\rho }_{970}$	[49]
Photochemical reflectance index	PRI-1	$({\rho }_{531}-{\rho }_{570}) / {(\rho }_{531}+ {(\rho }_{570}$)	[51]
Photochemical reflectance index	PRI-2	$({\rho }_{570}-{\rho }_{539}) / {(\rho }_{570}+ {\rho }_{539}$)	[68]
Red-green index	RGI	${\rho }_{690} / {\rho }_{550}$	[76]
Normalized difference water index	NDWI	${\rho }_{970} / {\rho }_{900}$	[49]
Carotenoids reflectance index-1	CARI-1	${\rho }_{510} /{\rho }_{550}$	[55]
Carotenoids reflectance index-2	CARI-2	$(1/{\rho }_{510}) / (1/ {\rho }_{700}$)	[55]
Plant senescence reflectance index	PSRI	$({\rho }_{680}-{\rho }_{500}) / {(\rho }_{750}$)	[77]
Normalized pigment chlorophyll index	NPCI	$({\rho }_{680}-{\rho }_{430}) / {(\rho }_{680}+ {(\rho }_{430}$)	[69]
Pigment specific simple ratio for chlorophyll-a	PSSR-b	${\rho }_{800 } / {\rho }_{650}$	[78]
Pigment specific simple ratio for chlorophyll-b	PSSR-a	${\rho }_{800} / {\rho }_{675}$	[78]
Anthocyanin reflectance index-1	ARI-1	$(1/{\rho }_{550}) / (1/ {\rho }_{700}$)	[57]
Anthocyanin reflectance index-2	ARI-2	${\rho }_{800} (1/{\rho }_{550}) / (1/ {\rho }_{700}$)	[57]
Structure insensitive pigment index	SIPI-1	$({\rho }_{800}-{\rho }_{450}) / {(\rho }_{800}+ {(\rho }_{650}$)	[58]
Structure insensitive pigment index	SIPI-2	${\rho }_{800}-{\rho }_{440}) / {(\rho }_{800}+ {(\rho }_{680})$	[58]

**Table 2:** Broad band vegetation indices for phenotyping specific traits in plants

Broad band VI	Abbreviation	Formula	Reference
Violet wavelength	VIO	${\rho }_{400} / {\rho }_{451}$	
Blue wavelength	BL	${\rho }_{454} to {\rho }_{496}$	
Green wavelength	GR	${\rho }_{499} to {\rho }_{517}$	
Yellow wavelength	YW	${\rho }_{574} to {\rho }_{589}$	
Orange wavelength	OR	${\rho }_{592} to {\rho }_{619}$	
Red wavelength	RED	${\rho }_{622} to {\rho }_{748}$	
Red-edge wavelength	RE	${\rho }_{691} to {\rho }_{730}$	
Near infrared wavelength	NIR	${\rho }_{751} to {\rho }_{997}$	
Normalized difference vegetation index	NDVI	$(NIR-Red) / (NIR+Red)$	[48]
Simple ratio index	SR	$NIR / Red$	[47]
Green normalized difference vegetation index	NDVI-green	$(NIR-Green) / (NIR+Green)$	[79]
Modified simple ratio	MSR	$Red/{ (NIR/Red+1)}^{0.5}$	[80]
Renormalized difference vegetation index	RDVI	$(NIR-Red) / {(NIR+Red)}^{0.5}$	[81]
Red-green vegetation index	RGRI	$Red-Green$	[82]
Ratio vegetation index	RVI	$Red / NIR$	[76]
Difference vegetation index	DVI	$NIR-Red$	[83]
SR & NDVI	SR-NDVI	$({NIR}^{2}-Red) / (NIR+{Red}^{2})$	[53]
NDVI-Red-edge	NDVI-RE	$(NIR-{Red}_{edge}) / (NIR+{Red}_{edge})$	[76]
Red_edge_ chlorophyll index	CI-Red_edge_	$(NIR / {Red}_{edge})-1$	[76]
Anthocyanin reflectance index	ARI-1	$(1/Green) / (1/{Red}_{edge})$	[76]
Modified Anthocyanins reflectance index	mARI	$\left(1/Green\right) / \left(1/{Red}_{edge}\right)*NIR$	[76]
Anthocyanin reflectance index	ARI-2	$Green / NIR$	(76)

The broad and narrow indices developed in this study are presented in Table 3. The selection of these indices was based on their high R^2^ in the cross validation (LOOCV) and low root mean square error (RMSE) estimates in the bootstrapping analysis. EWI-13 and EWI-14 estimated 65% and 62% of the EW variation combining the wavelengths 625, 736 and 832 nm. The indices EWI-6, EWI-9 and EWI-12 integrated only two wavelengths, 658 and 712 nm; 670 and 718 nm; and 622 and 718 nm, respectively. The proportion of the variance in the EW explained by the EWI (R^2^) was as follow: EWI-6 = 0.52, EWI-9 = 0.51 and EWI-12 = 0.51. When the broad spectral bands blue, red and NIR were combined in the spectral indices, the prediction accuracy ranged from 31 to 44%. Specifically, EWI-1 estimated 44% of the variability with a RMSE of 1.19 mg·dm^−2^. The slope of the linear models (B) in most cases was positive, except those for EWI-3, EWI-4, EWI-8, EWI-10 and EWI-11. The increase in EW content of 1 mg·dm^−2^ caused wide variations in the values of the broad and narrow indices from 0.002 to 5.73.

**Table 3:** Coefficients of determination (R^2^) and root mean square error (RMSE in mg·dm^−2^) of the indices developed for phenotyping EW in leaves

Index	Model parameters					
	a	B	R^2^	RMSE	95% CI	p-value
Broad indices / RGB and NIR spectral bands						
EWI-1 _Blue/Red_	0.213	0.04	0.44	1.19	1.037–2.17	< 0.0001
EWI-2 _Blue/NIR_	0.07	0.13	0.39	1.18	0.98–1.98	< 0.0001
EWI-3 _(NIR-Red)/Blue_	− 0.93	− 0.01	0.31	1.19	1.04–1.97	< 0.0001
EWI-4 _(Red_^2^_-Blue)/(Red-Blue_^2^_)_	− 0.09	− 0.03	0.32	1.19	1.09–2.55	< 0.0001
Narrow indices / two narrow spectral bands						
EWI-5 _676_	0.019	0.005	0.45	0.97	0.75–1.21	< 0.0001
EWI-6 _658/712_	0.12	0.03	0.52	1.02	0.70–1.36	< 0.0001
EWI-7 _625/706_	0.22	0.05	0.50	0.96	0.67–1.28	< 0.0001
EWI-8 _694/625_	− 0.006	− 0.002	0.42	1.08	0.96–1.55	< 0.0001
EWI-9 _(670–718) / (670+718)_	− 0.85	0.03	0.51	1.04	0.61–1.54	< 0.0001
EWI-10 _(691–661) / (691+661)_^2^	4.92	− 1.03	0.48	0.99	0.74–1.27	< 0.0001
EWI-11 _(1/661)—(1/694)_	29.13	− 5.73	0.48	1.01	0.71–1.35	< 0.0001
EWI-12 _(622/718)-1_	0.62	0.12	0.51	0.99	0.74–1.28	< 0.0001
Narrow indices / three narrow spectral bands						
EWI-13 _625 (1/736 – 1/832)_	0.008	0.004	0.65	1.01	0.622–1.426	< 0.0001
EWI-14 _(625–736) / 832_	0.02	0.007	0.62	0.98	0.65–1.35	< 0.0001

R^2^ was calculated in the training set by a leaving one out cross validation analysis (LOOCV) and the RMSE was estimated in the validation set. B is the slope of the line and a is the intercept of the dependent variable

### Prediction accuracy of spectral indices for phenotyping of EW load

The EW content determined with the chemical method from samples collected in the field experimental trials of the mapping population ranged from 1.54 to 2.4 mg·dm^−2^ (Table 4). The heritability estimate (h^2^) of EW ranged from 0.51 to 0.58 across all three trials. Overall, the CV of all trials was low, 6.9 in DS-1, 5.8 in DS-2 and 7.6 in DS-3. All fourteen indices included in Table 3 were calculated with ground spectral information. However, only the indices EWI-1, EWI-2, EWI-3, EWI-4, EWI-9 and EWI-13 were strongly associated with EW deposition when estimated with canopy reflectance and are the only indices included and discussed in Table 4.

**Table 4:** Mean, genetic variance (${{\varvec{\sigma}}}_{{\varvec{g}}}^{2}$), heritability estimate (${{\varvec{h}}}^{2}$), error variance (${{\varvec{\sigma}}}_{{\varvec{e}}}^{2}$) and coefficient of variation (CV in %) of EW content (mg·dm^−2^), EWI-1, EWI-2, EWI-3, EWI-4, EWI-9 and EWI-13

		Mean	${\sigma }_{g}^{2}$	${h}^{2}$	${\sigma }_{e}^{2}$	CV
EW (mg·dm^−2^)	DS-1	1.72	0.014	0.56	0.014	6.9
	DS-2	2.40	0.015	0.51	0.018	5.8
	DS-3	1.54	0.016	0.58	0.014	7.6
EWI-1	DS-1	0.31	0.00039	0.78	0.00025	5.0
	DS-2	0.37	0.0004	0.86	0.0001	2.9
	DS-3	0.41	0.0007	0.78	0.0004	5.2
EWI-2	DS-1	0.31	0.00039	0.83	0.00025	5.1
	DS-2	0.17	0.0003	0.77	0.0002	7.9
	DS-3	0.41	0.00001	0.46	0.0004	5.2
EWI-3	DS-1	6.72	2.07	0.85	0.72	12.6
	DS-2	3.2	0.34	0.78	0.19	13.7
	DS-3	26.6	7.4	0.48	16.5	13.9
EWI-4	DS-1	− 1.28	0.0006	0.58	0.0008	22.7
	DS-2	-0.23	0.0003	0.65	0.0002	6.8
	DS-3	− 0.37	0.0007	0.80	0.0003	5.3
EWI-9	DS-1	-0.48	0.0065	0.84	0.0024	10.2
	DS-2	− 0.28	0.0016	0.74	0.0011	12.1
	DS-3	− 0.12	0.00006	0.63	0.00007	7.3
EWI-13	DS-1	0.72	0.0001	0.75	0.00007	11.5
	DS-2	0.092	0.000039	0.78	0.000023	5.2
	DS-3	0.29	0.0001	0.62	0.0001	3.6

EW and the indices were estimated across three sets of data (DS 1 to 3) collected from wheat inbreed lines evaluated during the agronomic cycle from 2012 to 2013, 2014 to 2015 and 2016 in the research station of CIMMYT near at Ciudad Obregon, Sonora in Mexico and Bushland, Texas

**Fig. 3:**
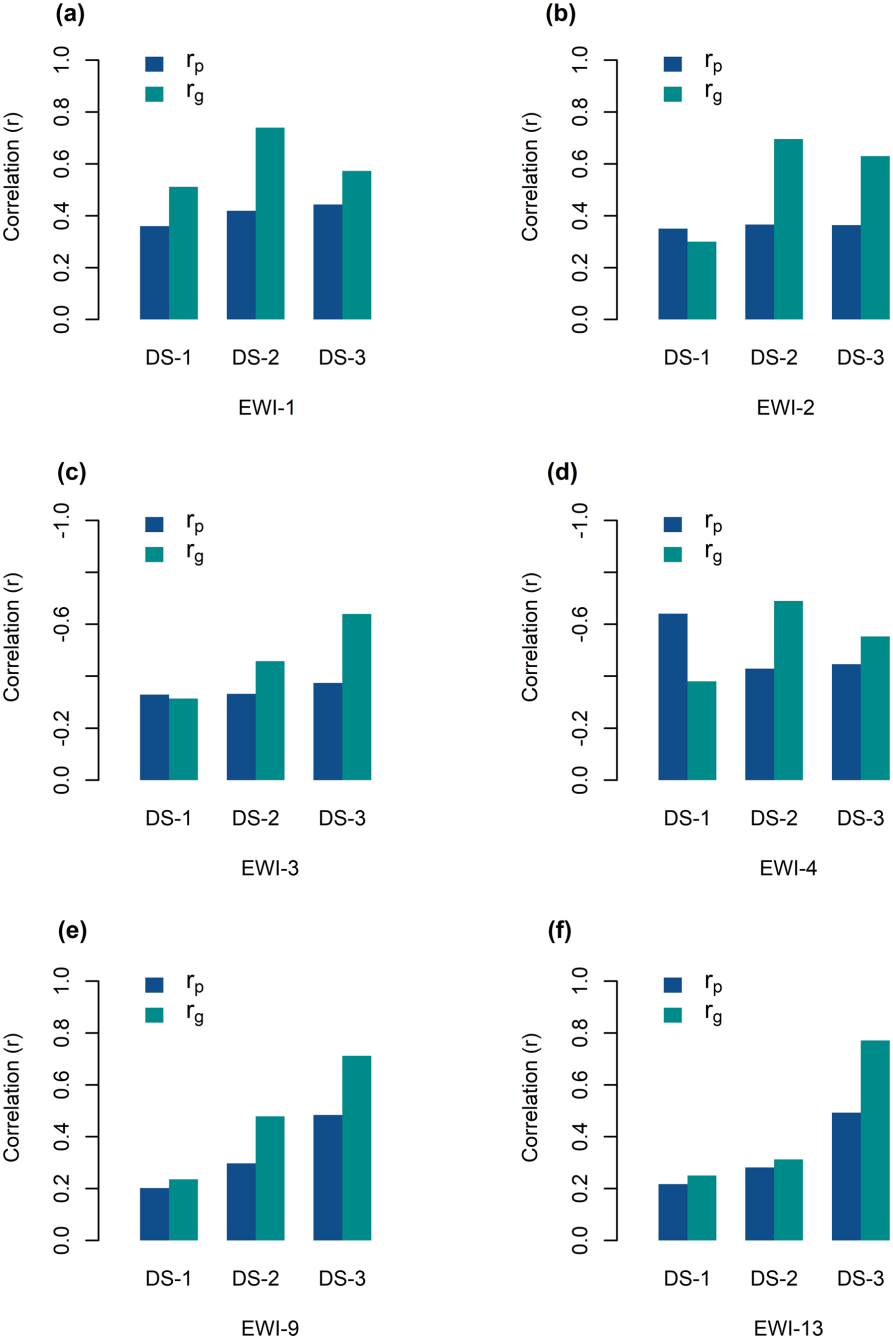
Phenotypic $({\sigma }_{p}$) and genotypic (${\sigma }_{g})$ correlation of the epicuticular wax indices (EWI) 1, 2, 3, 4, 9 and 13 with EW content measured with the chemical method (mg·dm^−2^). All six indices were statistically significant (*P* < *0.01*) across all three sets (DS-1, 2 and 3)

The phenotypic (r_p_) and genotypic (r_g_) correlations of the top performing indices estimated with the ground hyperspectral information and EW content are presented in Fig. 3. All correlations were statistically significant at *P* ≤ *0.01*. As expected, genotypic correlations were higher than phenotypic correlations in all cases. According to these parameters, the index EWI-4 and EWI-9 better estimated the variation of EW. The average response of r_p_ and r_g_ were -0.51 and -0.55 for the EWI-4, and 0.33 and 0.48 for EWI-9.

Although the lack of variance of EW in the mapping population used in this study might limit the response for direct selection, the moderate h^2^ of the trait would lead to genetic advances when selection is applied (Table 5). The genetic gain (GG) for EW with selection pressure of 10% is also included in Table 5. In DS-1, the GG was 0.65 mg·dm^−2^; in DS-2, it was 0.89 mg·dm^−2^, and in DS-3 it was 0.59 mg·dm^−2^. However, when the mean of the actual population was considered, the genetic advance with direct selection averaged 2.5%. The correlated response of the EW indices with the EW content derived an increase in EW. Improvement in the EWI-1, 2, 9 and 13 resulted in increases of 0.063, 0.053, 0.047 and 0.043 mg·dm^−2^ of EW content, respectively. On the other hand, decreases of 0.047 and 0.063 mg·dm^−2^ were calculated with a positive selection of the indices EWI-3 and 4. The efficiency of selection (RE) based on the secondary characters or indices (EWI) ranged from 46 to 78% in average. However, EWI-1, 2 and 4 in DS-2 were almost as efficient in selection as the direct selection of the trait with the chemical method with 112, 99 and 90% efficiency, respectively.

**Table 5:** Genetic gain (GG in mg·dm^−2^), genetic advance with respect to the mean (GAM in %) and response to direct selection (R) of EW

		DS-1	DS-2	DS-3
GG	EW	0.65	0.89	0.59
GAM (%)	EW	2.5	1.7	3.3
R	EW	0.09	0.08	0.12
CR	EW & EWI-1	0.05	0.08	0.06
CR	EW & EWI-2	0.03	0.08	0.05
CR	EW & EWI-3	− 0.03	− 0.05	− 0.06
CR	EW & EWI-4	− 0.06	− 0.07	− 0.06
CR	EW & EWI-9	0.02	0.05	0.07
CR	EW & EWI-13	0.02	0.03	0.08
RE	EW & EWI-1	0.65	1.12	0.55
RE	EW & EWI-2	0.46	0.99	0.46
RE	EW & EWI-3	− 0.41	− 0.66	− 0.48
RE	EW & EWI-4	− 0.71	− 0.90	− 0.53
RE	EW & EWI-9	0.27	0.67	0.62
RE	EW & EWI-13	0.26	0.45	0.66

Correlated response (CR) of EW content and indices, and relative efficiency of indirect selection (RE) of EW with indices 1, 2, 3, 4, 9, and 13

### Multivariate regression models integrating narrow spectral bands to predict the EW load

The statistics of the multivariate models developed with the selected bands in the PLSR analysis are presented in Table 6. The final selection of the variables led to seven models in single and/or multiple combinations of eight wavelengths. The spectral response at 424 nm predicted almost 33% of the total variability of the trait in the validation set. However, when as many as seven spectral bands were incorporated in a model (424, 547, 574, 658, 712, 721, 775 and 817 nm), the accuracy increased by 38% (EWM-7 with R^2^ = 0.71). The RMSE of the prediction was consistent across the models, ranging from 0.49 to 0.52 mg·dm^2^.

**Table 6:** Statistics of regression models (EWM)

		Spectral band in nanometers								RMSE	R^2^
**EWM-1**	**Intercept**	**424**								0.49	0.33
	0.46	52									
**EWM-2**	**Intercept**	**658**	**721**							0.50	0.45
	0.31	90.3	− 3.88								
**EWM-3**	**Intercept**	**712**	**721**	**775**	**817**					0.51	0.58
	0.71	160.7	− 183.1	− 33.7	80.2						
**EWM-4**	**Intercept**	**658**	**712**	**721**	**775**	**817**				0.51	0.60
	0.19	40.8	109.6	− 128.4	− 65.8	99.1					
**EWM-5**	**Intercept**	**574**	**658**	**712**	**721**	**775**	**817**			0.51	0.61
	-0.22	17.3	16.9	82.9	− 109.1	− 122.2	153.4				
**EWM-6**	**Intercept**	**424**	**574**	**658**	**712**	**721**	**775**	**817**		0.52	0.66
	1.13	− 76.4	1.9	134.7	76.4	− 109.9	− 45.1	78.1			
**EWM-7**	**Intercept**	**424**	**547**	**574**	**658**	**712**	**721**	**775**	**817**	0.52	0.71
	− 3.1	− 73.5	58.9	− 82.7	146.9	89.6	− 188.8	− 91.6	134.3		

The coefficient of determination (R^2^) and the C(p) were calculated in the training set, while the root mean square error (RMSE) was estimated in the validation set. The multivariate models were significant at 5% of probability or less

Bold values indicate the parameters of the statistical model: intercept and wavelength range in nanometers (nm)

**Fig. 4:**
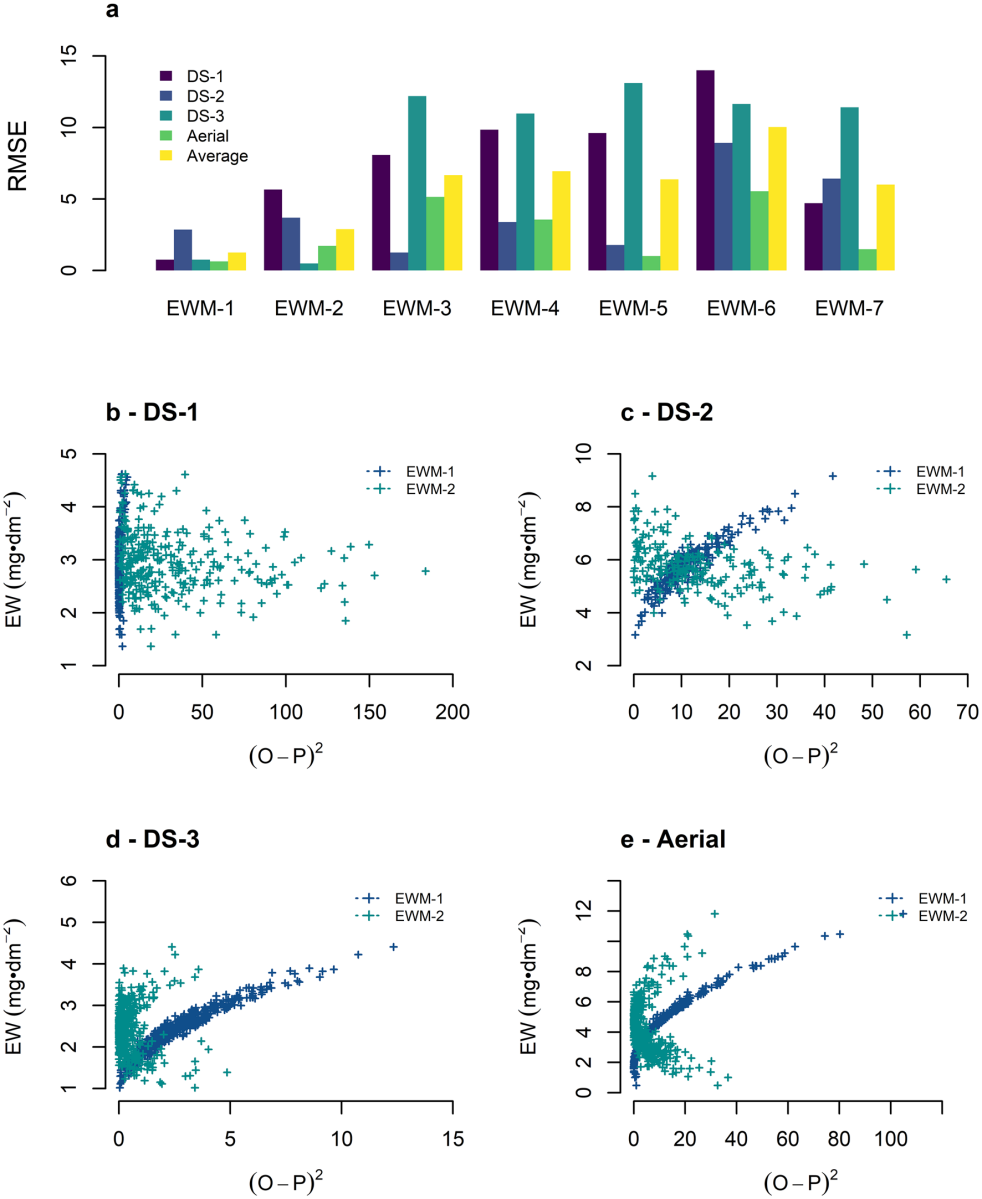
Root Mean Square Error (RMSE in mg·dm^−2^) of the multivariate models (EWM) for predictions of epicuticular wax load utilizing the ground-based and aerial hyperspectral reflectance. The RMSE of prediction for the ground-based information is presented as the average response across the four sets of wheat inbreed lines evaluated. DS stands for data set 1 to 3 and Aerial corresponds to the hyperspectral information collected with the aircraft. (O-P)^2^ is the square of the difference between the observed minus the predicted values

The RMSE of the seven EW models was estimated in the four experimental trials in which ground and aerial reflectance were collected. The square root of the residuals is presented in Fig. 4a. A considerable increase in the error of the prediction models calculated with ground and aerial hyperspectral information was observed across all trials in comparison to the RMSE estimated with reflectance from single leaves. The highest prediction accuracy was obtained with EWM-1, with an average RMSE of 1.4 mg·dm^−2^ from the ground measurements and of 0.63 mg·dm^−2^ from the aerial information. EWM-2 seems to accurately estimate EW load utilizing light reflectance in the same way as EWI-1. However, in cases as DS-1, the prediction accuracy with the EWM-2 led to a RMSE as high as 5.7 mg·dm^−2^.

## Discussion

In this study we evaluated the spectral response of leaves and derived and validated a set of indirect methods for phenotyping the trait. Furthermore, differences in light interaction derived by cuticular waxes and detected in this study coincide with results from studies conducted in *Vitis vinifera* [59], *Leucadendron lanigerum* [60] and *Cotyledon orbiculata* [61]. The increase of approximately 10% of light reflectance in the NIR region was considerably less than changes in reflectance previously detected on wheat ~ 15% [62] and in rosette succulent plants ~ 50% [31]. Additionally, the violet (r = 0.64) and blue (r = 0.63) spectral regions strongly correlated with EW, and it is in line with preliminary studies where waxes were reported as photoprotective mechanisms against short wavelength radiation [17, 63–65]. A significant number of research studies also reported that waxes enhance UV (~ 100 to 400 nm) reflectance [17, 28, 63, 66], but the analysis of these wavelengths is outside of the scope of this study due to limitations of the equipment utilized to collect the spectral information.

The wax cuticle enhances light reflectance almost by 1% per every unit of wax (mg·dm^−2^) accumulated on top of the leaf surface, but specifically in the PAR wavelengths where the absorption of photosynthetic pigments occurs [17]. In sorghum (*Sorghum bicolor* L.), a similar increase of 3% in reflectance by the cuticular leaf coat was reported, but the result was based on wavelengths of the spectrum from 400 to 1000 nm, without a detailed examination of specific narrow spectral bands [67]. Among the spectral regions associated with EW, the wavelength at 424 and 448 nm are linked to the absorption of light by carotenoids in plants [56], while the surrounding wavelengths at approximately 500 nm are associated with the dissipation of excess radiation and the efficiency of photosynthetic radiation [50, 68, 69]. Additional peaks of absorption of chlorophyll a and b in the 600 nm coincides with two main wavelengths linked to EW in 625 and 660 nm.

Several narrow and broad indices for phenotyping additional traits in plants were correlated with EW load, but the moderate to low correlation (Table 7) of these indices with EW limits any form of application for phenotyping. Although a reasonable r^2^ value of 0.65 was estimated when three narrow spectral bands were integrated in the novel spectral indices (EWI-13 and EWI-14 in Table 2), the high cost of sensors required to acquire hyperspectral reflectance can limit the utilization of these indices. On the contrary, EWI-1 requires two main spectral ranges (blue and red) that can easily be extracted from RGB images. Broad-sense heritability was estimated for the EW indices and the EW measured by the chemical method (Table 4). In all three trials h^2^ was considerably higher than in prelaminar published results [70]. The six EW indices enabled a more reliable and precise quantification of the proportion of the genetic variance of the trait by considerably decreasing the error variance. However, the coefficients of variation of the indices EWI-3, EWI-4, EWI-9 and EWI-13 in DS-1 were considerably large due to the dispersion of the data around the mean of the population.

**Table 7:** Correlation of the narrow and broad vegetation indices and the EW content (mg·dm^−2^)

Narrow vegetation indices	Correlation coefficient (r)	Broad vegetation indices	Correlation coefficient (r)
Water index	− 0.18^ns^	Violet wavelength	0.64^b^
Photochemical reflectance index-1	− 0.57^b^	Blue wavelength	0.63^b^
Photochemical reflectance index-2	0.41^b^	Green wavelength	0.48^b^
Red-green index	− 0.08^ns^	Yellow wavelength	0.49^b^
Normalized difference water index	− 0.45^b^	Orange wavelength	0.52^b^
Carotenoids reflectance index-1	0.46^b^	Red wavelenght	0.33^a^
Carotenoids reflectance index-2	− 0.67^b^	Red-edge wavelenght	− 0.006^ns^
Plant senescence reflectance index	− 0.31^a^	Near infrared	0.39^b^
Normalized pigment chlorophyll index	− 0.44^b^	Normalized difference vegetation index	0.08^ns^
Pigment specific simple ratio for chlorophyll-a	− 0.57^b^	Simple ratio index	0.11^ns^
Pigment specific simple ratio for chlorophyll-b	− 0.55^b^	Green normalized difference vegetation index	− 0.34^a^
Anthocyanin reflectance index-1	− 0.52^b^	Modified simple ratio	0.28^a^
Anthocyanin reflectance index-2	− 0.58^b^	Renormalized difference vegetation index	0.31^a^
Structure insensitive pigment index-1	− 0.34^b^	Red-green vegetation index	− 0.57^b^
Structure insensitive pigment index-2	− 0.61^b^	Ratio vegetation index	− 0.08^ns^
		Difference vegetation index	0.37^**^
		Simple ratio and normalized difference vegetation index	0.33^a^
		Normalized difference vegetation index- Red-edge	0.42^b^
		Red_edge_ chlorophyll index	0.43^b^
		Anthocyanin reflectance index-1	− 0.67^b^
		Modified Anthocyanins reflectance index	− 0.45^b^
		Anthocyanin reflectance index-2	0.32^a^

*ns* not significant; ^a^ and ^b^Significant at 5% and 1% probability, respectively

The moderated h^2^ of EW let to genetic gains of up to 3%, a reasonable advancement for a quantitative trait and superior to genetic gains of ~ 1% in grain yield [71, 72]. All four broad and two narrow indices presented in Table 5 positively improve EW content, except EWI-3 and EWI-4, for which negative selection is needed to increase the EW load on leaf surfaces. The efficiency of indirect selection with the spectral indices was highly dependent on the experimental trial and its coefficient of variation, as it is the case in the DS-2 where selection with the EWI-1 was 12% more efficient that the direct selection. Examining the residuals of the model against the EW measurements, we observed a shift towards an increase in the residuals of EWI-1 as the EW content increases, suggesting a potential restriction on utilizing this index for phenotyping genotypes where the EW is above 6 mg·dm^−2^. However, this was not observed with the residuals of EWM-2. We suspect that implementing the EWI developed in this study with aerial spectral reflectance might lead to a low-quality phenotyping of EW and could potentially lead to confounding results.

## Conclusions

EW is the outermost cuticle of leaves and directly affects light interactions, especially reflectance. This cuticle increases light reflectance at the visible and NIR regions by 0.5% and 1.6%, respectively. Integrating specific narrow wavelengths that are highly sensitive to variations in the EW load, we generated several spectral linear models and vegetation indices for predicting the EW content and detecting cultivars with low and high EW. The prediction accuracy of these phenotyping methods was dependent on the characteristics of the sensor utilized to capture the spectral information, as well as on the canopy architecture and the distance of the sensor from the ground. With light reflectance captured from either the adaxial or abaxial side of the leaf, the broad index EWI-1 and the narrow index EWI-13 can accurately estimate EW. However, for canopy reflectance, the indices EWI-4 and EWI-9 more accurately estimate the density of the cuticle and led to a similar genetic advance than that from direct selection for the trait. The multivariate regression model EWM-7 integrated eight wavelengths distributed across the visible and NIR spectra and estimated 71% of the variation of the trait from the reflectance of a single leaf. In contrast, with ground and aerial reflectance, EWM-1 and EWI-2 accurately estimated the EW content (mg·dm^−2^), but insensitivity to variation at EW values larger than 6 mg·dm^−2^ was detected for EWI-1.

## Methods

### Plant material and culture

The first set of genotypes evaluated were twenty-four recombinant inbred lines (RILs) derived from a cross of the spring cultivars Halberd (tolerant to heat stress) and Len (susceptible to heat stress). The lines were grown in a completely randomized design (CRD) with four replications in a growth chamber programmed with intervals of twelve hours of light and dark. Plants were sown in nursery pots 0.185 m in height with a diameter of 0.162 m that were filled with peat moss. The plants were fertilized twice during the growing season with the standard fertilizer 20–20-20 (N-P_2_O_5_-K_2_O).

### Leaf radiometric measurements

The spectral response from 350 to 1050 nm was captured with a CI-710 miniature leaf spectrometer from CID Bio-Science in 3022 spectral channels. The equipment was calibrated every five minutes with an integrated BaSO4 white reference disk for 100% reflectance and a black panel for 0% reflectance. Ten readings of the light reflected by the flag leaf were obtained prior to collecting the leaf sample for wax quantification. The spectral range of the signatures were adjusted to 400–900 nm and the spectral resolution to 3 nm by averaging the spectral channels included every 3 nm. The last step was to estimate the average of the ten spectral readings, only the averaged signature was considered for further analysis.

### Quantification of epicuticular wax

Leaf samples were collected after the light reflectance at the adaxial and abaxial sides of the leaf was recorded, approximately 10 days after pollination (DAP). Each sample consisted of six leaf punches of 0.01 m diameter and were collected in 2.0 ml (ml) glass vials. The EW was extracted by immersing the leaf punches in 1.5 ml of HPLC chloroform (CHCl_3_) for 20 s, and the EW was quantified via the colorimetric method described by [14]. The optical density of every sample at 590 nm was measured with PHERAstar® spectrophotometer. A standard curve was developed to transform the readings of absorbance to milligrams (mg) per square decimeter (dm^2^) of EW.

### Partial least square (PLSR)

A supervised multivariate model was built to predict EW (*Y*) in a training set of data by applying the partial least square regression (PLSR) approach. PLSR is a statistical method that combines the theoretical principles of multiple linear regression and principal component analysis (PCA) to address the situations where several highly correlated predictor variables and relatively fewer samples exist. This approach decomposes the response variables (*X*) into orthogonal scores (*T)*, loadings (*P*) and the error (*E*) while simultaneously incorporating the information from the variables:

1 $$X = TP' + E$$

Two hundred spectral bands were integrated in the PLS analysis to identify a set of components that best estimated EW content. The RMSE (root mean square error) of the prediction was estimated with a leave-one-out cross-validation analysis (LOOCV) in a subset of the data with 66.7% of the observations. The EW content was predicted in the remaining 33% of the observations (validation set), integrating the optimum number of components detected in the PLSR model. The analysis was conducted with the *plsr* function included in the *pls* package in the statistical software R [73].

### Narrow and broad empirical spectral indices for the indirect estimation of the EW content of leaves

Vegetation indices developed to phenotype the morphological and physiological characteristics of the plant (Tables 1 and 2) were calculated with the light reflectance. The correlation coefficients and the statistical significance of each of the VIs and the EW content were estimated with the *cor* function in the statistical software R.

Spectral indices were calculated by combining the light reflectance at different wavelengths. Eleven mathematical combinations of the spectral bands were calculated including the reflectance at every 3 nm. The adjustment of the spectral resolution was done by averaging the percentage of light reflectance every 3 wavelengths. Additional combinations were also calculated with the average light reflectance of the spectral range of the blue, green, yellow, red and NIR regions (Table 2). In each of the mathematical combinations one, two and up to three bands were integrated. The significance of the linear models and the coefficient of determination (R^2^) of every pairwise combination of the spectral bands was calculated with the *lm* function in a leave one out cross-validation (LOOCV) analysis. The format of the linear models was the follow:

2 $${y_i} = {\beta _0} + {\beta _1}\left( {{x_i}} \right)$$

where ${\mathrm{y}}_{\mathrm{i}}$ corresponds to the EW content (mg·dm^−2^), ${\mathrm{x}}_{\mathrm{i}}$ is the value of the spectral index, and ${\upbeta }_{0}$ and ${\upbeta }_{1}$ are the intercept and the slope of the model, respectively. A set of indices were selected based on the highest R^2^ criteria in a LOOCV analysis. An inverse prediction approach was applied for assessing the predictive capability of the indices selected. These selected indices were fitted in a calibration data set (66% of the observations) using the same format as in Eq 2 but with ${y}_{i}$ as the spectral index and ${x}_{i}$ the EW content [74, 75]. The step after the models were fitted was to solve for ${x}_{i}$ with the estimates of the parameters calculated in the training data set with the follow equation: ${\mathrm{X}}_{\mathrm{i}}=\frac{{\mathrm{y}}_{\mathrm{i}}-{\upbeta }_{0}}{{\upbeta }_{\mathrm{i}}}$. Estimates of the RMSE were bootstrapped 1000 times for each of indices and in the remaining 34% of the observations with the estimated values of EW with the equation and the chemically measured EW content.

### Stepwise regression (SWR)

The spectral bands statistically associated with the EW content (Figs. 1 and  2) were incorporated to build a multivariate model for prediction. These variables were included and/or removed based on the significance of the partial F-values. The final models were selected when the inclusion of more spectral bands was statistically not justifiable. This analysis was conducted with the PROC REG statement in the statistical analysis software SAS [84] in a random training set of the total data (60% of the observations). The prediction models were selected based on the Mallows’ Cp estimator. The estimate of the RMSE of each prediction model was calculated in the remaining 40% of the observations, and final models were choosen based on the lowest value of the RMSE.

### Plant material and field experimental trials for validation

Two panels of spring wheat cultivars were evaluated during the agronomic cycle in 2013 at the Norman E. Borlaug Experimental Station (CENEB), Ciudad Obregon, Sonora in northwestern Mexico (27.20°N, 109.54°W, 38 masl). The panels were two sets of 114 and 216 landraces and product of interspecific hybridization with wild relatives. These experimental trials were established as an alpha-lattice design with two replications in a raised bed system with two rows per bed and were planted 80 days later than the normal planting date of wheat in the Yaqui Valley. Late panting allowed the genotypes to be exposed to average daily temperatures of 28 °C and maximum environmental temperatures of 39 °C during the heading and anthesis stages of the crop. There was an intern row spacing within each bed of 10 cm (cm), and a space between beds of 80 cm. In 2016, an additional panel of synthetic derived wheat lines (SDLs) was also evaluated in Bushland and College Station, Texas under non-irrigated conditions. The panel of SDLs were established in an alpha-lattice design with two replications and a plot size of 3.0 × 1.5 m.

### Ground base radiometric measurements for the direct validation

The canopy reflectance was collected with a FieldSpec 4 Hi-Res spectroradiometer that captured the light reflected in 2151 continuous bands with a spectral resolution of 3 nm (nm) from 0.35 to 0.7 µm and 8 nm from 1.4 to 2.1 µm. The measurements were collected from 11 AM to 1 PM by placing the optic fiber of the spectroradiometer 40 cm (cm) above the canopy. The sensor was radiometrically calibrated with a white BaSO4 reference card for 100% reflectance and by blocking the light intercepted by the optic fiber for 0% reflectance. Ten readings per plot were captured and the average response of these signatures at a single wavelength was utilized in further analysis.

### Airborne hyperspectral information

A set of aerial hyperspectral images were captured from the panel of wheat SDLs in College Station, Texas. The images were obtained with an Aisa KESTREL-10 hyperspectral camera, developed by SPECIM®, and mounted on a Cessna 355 II aircraft. An altitude of 5000 feet (ft) and a speed of 192 km per hour (km/h) were maintained through the flight of the aircraft. The camera captured 120 spectral bands with spectral and spatial resolutions of 5 nm and 0.25 m, respectively. For calibration, four 8 m by 8 m ground tarps with nominal reflectance values of 8%, 16%, 32% and 48% were laid out in the field and captured in the hyperspectral images. The exact percentage of reflectance of the tarps was captured with a Hand-held 2 spectroradiometer. The range of this spectroradiometer is from 0.325 to 1.075 µm, and the spectral resolution is 3 nm. The hyperspectral images were georeferenced and ensembled using the image analysis software ERDAS®. Digital counts (DCs) were extracted individually for each tarp and for individual plots with the software ENVI®. A linear regression model for a single spectral band was developed using light reflectance captured with the spectroradiometer from the tarps as the response variable (dependent) and the DCs as the independent variable. The linear equations were utilized for the estimation of the total canopy reflectance of each of the two hundred spectral bands in each plot.

### Efficiency of indirect selection of EW with spectral information

The fourteen spectral indices (EWI) and the eleven regression models developed in this study (Table 4) were calculated with the ground based and aerial spectral information collected in the four experimental trials. Each of the indirect selection methods (spectral indices and models) was considered as an independent variable and subjected to an analysis of variance (ANOVA) for an alpha-lattice experimental design with the *lmer* function included in the package lme4 in the statistical software R. The variance components were extracted with the function *varComp* and estimates of the heritability in a broad sense (${\mathrm{h}}^{2})$ calculated according to the formula described by [85]: ${\mathrm{h}}^{2}=\frac{{\sigma }_{g}^{2}}{{\sigma }_{g}^{2}+({\sigma }_{e}^{2}/r)}$ where ${\sigma }_{g}^{2}$ corresponds to the genetic variance, ${\sigma }_{e}^{2}$ to the error variance and *r* is the number of replications in the experimental trial.

The statistical relationship of the target trait (phenotypic correlation) and the spectral method was calculated with the *cor* function of the stats package, while the genetic relationship of the traits (genotypic correlation) was estimated with the following equation: ${\upsigma }_{\mathrm{g}}=\frac{{\mathrm{COV}}_{\mathrm{XY}}}{\surd {\mathrm{Var}}_{\mathrm{x}}{\mathrm{Var}}_{\mathrm{y}}}$, where COV_XY_ corresponds to the covariance estimate of the EWI and EW content calculated with the chemical method, Var_x_ is the variance of the EWI and Var_y_ is the variance of EW [86]. The COV_XY_ was calculated with the *cov* function, and Var_x_ and Var_y_ with the *varComp* function in R. The genetic correlation of the traits helps us to understand the pleiotropic action of the genes controlling the trait and its indirect selection.

The genetic gain (GG), the genetic advance (GA), the genetic advance with respect to the mean (GAM), the expected response to selection (R), the correlated response to selection (CR), the relative efficiency of indirect selection (RE) were all calculated according to Falconer [87]. GG, GAM, R, CR and RE were estimated as follows:

$\mathrm{GG}={\mathrm{h}}^{2}*\mathrm{SDiff}$, where ${\mathrm{h}}^{2}$ is the estimate of the broad sense heritability of the trait, and SDiff is the selection differential of the trait (EW) with a selection pressure of 10% ($\mathrm{SDiff}={\overline{\mathrm{x}} }_{\mathrm{p}}-{\overline{\mathrm{x}} }_{\mathrm{S}}$).

$\mathrm{GA}=\mathrm{K}\left({\upsigma }_{\mathrm{p}}\right){\mathrm{h}}^{2}$ where K is the selection differential, ${\upsigma }_{\mathrm{p}}$ is the phenotypic standard deviation of every spectral index or prediction model, and ${h}^{2}$ corresponds to the broad sense heritability. The k was estimated for 10% selection intensity as $\mathrm{k}=\overline{{\mathrm{x}}_{\mathrm{p}}}-\overline{{\mathrm{x}}_{\mathrm{s}}}$, where $\overline{{\mathrm{x}}_{\mathrm{p}}}$ and $\overline{{\mathrm{x}}_{\mathrm{s}}}$, are the population mean and the mean of the selected individuals, respectively.

$\mathrm{GAM }\left(\mathrm{\%}\right)=\frac{\mathrm{GA}}{\overline{\mathrm{x}}}\mathrm{ x }100$, where $\overline{\mathrm{x}}$ is the grand mean of the specific character.

$\mathrm{R}={\mathrm{h}}_{\mathrm{x}}{\upsigma }_{\mathrm{x}}$, where ${\mathrm{h}}_{\mathrm{x}}$ is the square root of the heritability and ${\sigma }_{x}$ is the genotypic standard deviation.

$\mathrm{CR}={\mathrm{h}}_{\mathrm{x}}{\mathrm{r}}_{\mathrm{gx}}{\upsigma }_{\mathrm{gy}}$, where ${\mathrm{h}}_{\mathrm{x}}$ is the square root of the heritability for trait X (spectral index), ${\mathrm{r}}_{\mathrm{gx}}$ is the genetic correlation of the spectral index and EW, and ${\upsigma }_{\mathrm{gy}}$ is the genotypic standard deviation of trait Y (EW).

$\mathrm{RE}=\frac{\mathrm{CR}}{\mathrm{R}}$, where CR is the correlated response to selection and R is the expected response to selection for the trait.

## Data Availability

The data sets generated and analyzed during the current study are available in the CIMMYT Publications Repository, https://data.cimmyt.org/dataset.xhtml?persistentId=hdl:11529/10548539. Correspondence should be addressed to camarillo.castillo.f@gmail.com.

## Abbreviations

EW: Epicuticular wax
EWI: Epicuticular wax index
EWM: Epicuticular wax model
PT: Physiological traits
GY: Grain yield
LI: Light interception
RUE: Radiation use efficiency
WUE: Water use efficiency
CT: Canopy temperature
EWM: Epicuticular wax model
NIR: Near infrared radiation
GG: Genetic gain
RMSE: Root mean square error
CV: Coefficient of variation
CRD: Completely randomized design
PLSR: Partial least square regression
PCA: Principal component analysis
EWM: Epicuticular wax model
LOOCV: Leave-one-out cross-validation
SWR: Stepwise regression
R: Response to selection
CR: Correlated response
RE: Relative efficiency of indirect selection
GA: Genetic advance
GAM: Genetic advance respect to the mean
